# Comparative Analysis of Facial Injuries From Electric and Gas Stoves: A National Electronic Injury Surveillance System (NEISS) Database Study

**DOI:** 10.7759/cureus.91499

**Published:** 2025-09-02

**Authors:** Aditi Patel, Samreen Shah, Jedidiah Lim, David Kang, Vishveshvar Ramkumar, Allison Brown, Randolph S Devereaux, Hassan Y Ebrahim, Zakaria Y Abd Elmageed

**Affiliations:** 1 Biomedical Sciences, Discipline of Pharmacology, Edward Via College of Osteopathic Medicine, Monroe, USA; 2 Biomedical Sciences, Discipline of Preventive Medicine, Edward Via College of Osteopathic Medicine, Monroe, USA

**Keywords:** electric stove, facial injuries, gas stove, household appliance safety, neiss database, pediatric burn injuries, stove-related injuries

## Abstract

Injury from household stoves can be a significant public health issue, with the potential to cause severe harm from both electric and gas stoves. This study aims to compare the prevalence and types of facial injuries with other types of body injuries, as well as age-and gender-related injuries, resulting from electric and gas stoves, using data from the National Electronic Injury Surveillance System (NEISS) database. Data from NEISS showed that gas stove incidents resulted in 102 facial injuries, accounting for 46.2% of all gas stove-related injuries by body part. In contrast, electric stoves were associated with 349 hand-related injuries, comprising 60.6% of all electric stove-related injuries. Further analysis revealed that children up to five years old are disproportionately injured by stoves, totaling 315 cases, which represents more than six-fold the number reported in the six to 10-year age group (n=47). It was found that boys experienced a peak in stove-related burns in 2020, likely attributed to the American lockdown due to the COVID-19 pandemic. The remaining burn injuries remained relatively stable over the 10 years. The higher incidence of gas stove-related facial injuries can be explained by the long-range burning effect of flames produced by gas stoves. Meanwhile, the hand-related injuries from electric stoves can be attributed to the curiosity of young children using their hands to explore their environment. The findings indicate that implementing advanced stove-safety features, along with proactive measures by households and healthcare providers, can substantially reduce stove-related injuries and foster a safer living environment for all members of the household.

## Introduction

The top layer of the skin, termed the epidermis, provides a necessary barrier against exogenous substances found in the environment while preventing dehydration through the regulation of fluid loss. The cell types found in the epidermis include keratinocytes, melanocytes, and the Langerhans cells, all of which provide structure, pigmentation, and immune surveillance for the epidermis [1]. Beneath the epidermis lies the dermis, consisting of collagen and elastin that preserve structural integrity, as well as the vascular plexus, which provides nutrition for all layers of the skin [2]. When the skin is injured from burns, it can lead to a physiological imbalance, which can lead to major disability or even death [1]. It is estimated that the incidence of burn injuries in the United States is between 140-215 per 100,000 [3]. The World Health Organization further reports that approximately 40,000 American citizens will require hospitalization for burns annually, underscoring the significant burden of burn injuries as a public health concern.

According to the 2019 National Burn Repository, while less than 3% of household accidents are due to fire, flame burns remain the leading cause of burn injuries in the United States [4]. Injuries resulting from household appliances pose a significant public health concern, with kitchen stoves being a primary source of these incidents [5]. Both electric and gas stoves present substantial risks due to their frequent use and potential for causing severe injuries [6]. During the COVID-19 pandemic, the American people were asked to maintain physical distance from other citizens and remain quarantined in their homes. This led to people spending a greater amount of time in their homes and presumably an increased amount of time in the kitchen [7]. Understanding the prevalence and nature of injuries associated with these appliances is critical for improving safety standards and reducing injury rates. In 2019, the total economic burden of injuries in the US, including those related to household stoves, was estimated at $4.2 trillion. Previous research has suggested varying injury profiles between these electric and gas stoves. Electric stoves are often linked to burns and scalds, while gas stoves are associated with a broader range of injuries, including cuts, lacerations, and thermal burns [5].

Therefore, this study seeks to compare the prevalence of facial injuries with other types of body injuries, as well as age-related and gender-related injuries, resulting from electric and gas stoves. By leveraging data from the National Electronic Injury Surveillance System (NEISS) database [8], this study seeks to clarify the prevalence and distinct patterns of injuries-such as facial or hand burns-linked to electric and gas stoves, with a focus on the underlying mechanisms contributing to these incidents.

## Materials and methods

Data collection

Available data from 2014 to 2023 were sourced from the NEISS, a national database that reports anonymous hospital cases of product-related injuries involving children and adults of all ages in the US [8]. This data includes cases from both private and non-private emergency hospitals, specifically focusing on injuries related to gas and electric stoves. The criteria for electric (n=576) and gas (n=221) stove incidents ranged from counter-mounted cooking surfaces to ovens and heating ranges. The data were then refined to include all age ranges and locations of injury from electric or gas stoves. The data was presented in stacked-cluster format to highlight the percentages of injuries associated with electric and gas stoves. The data were accessed in April 2024.

Statistical analysis

Statistical analysis was completed using JASP Team (2024) JASP (Version 0.95.0) (Computer software). A contingency table was created to summarize the number of burn injuries by age group and stove type. Age groups were constructed at five-year intervals from 0 to 90 years of age (e.g., 0-5, 6-10), and the stove type was characterized as either electric or gas. Rows in the table included the age ranges, and two columns represented the frequency of burns associated with each type of stove. The data were analyzed using a Chi-Square test of independence to determine if the distribution of burn frequency differed by type of stove across the categories of age groups.

## Results

**Figure 1:**
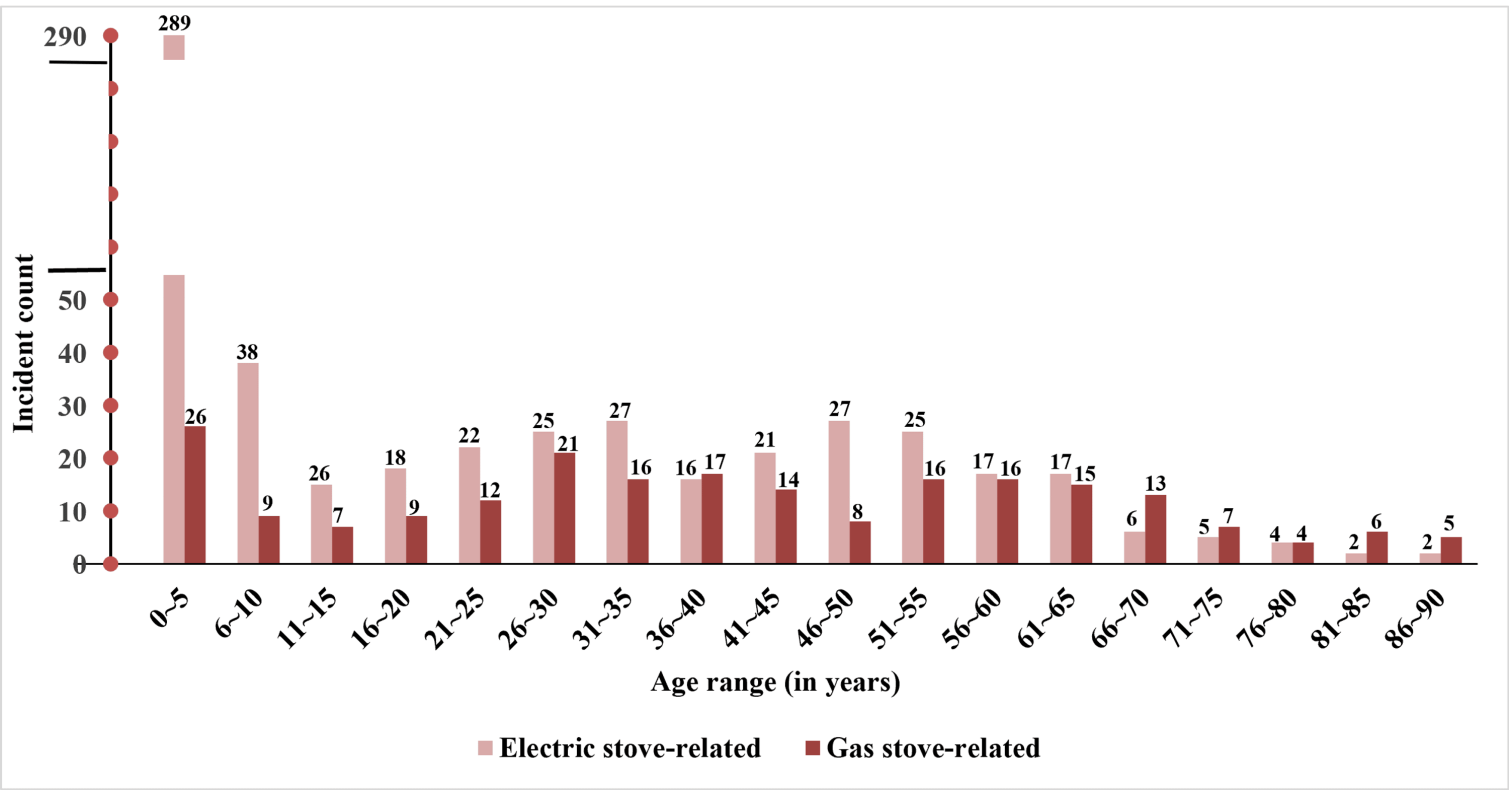
The number of electric and gas stove-related burn injuries in five-year age ranges, up to 90 years The data was gathered for all locations and involved burn injuries from both electric (n=576) and gas (n=221) stove-related incidents.

The total number of stove-related injuries reported in the NEISS from 2014 to 2023 was categorized into five-year periods, covering individuals from birth to 90 years of age. The highest incidence of stove-related gas injuries occurred in the newborn to five years age group, with 26 cases (11.8%), followed by another peak in the 26-30 age group with 21 cases (9.5%). A substantial decrease was observed among those over age 70, with only four cases (1.8%) reported among the 76-80 age group (Figure 1).

Similarly, electric stove-related injuries were most frequent in the newborn to five years age group, with 289 cases (40.9%), followed by a smaller number of cases in the range of 66-90 years old, with a total of 19 cases (3.3%).

**Figure 2:**
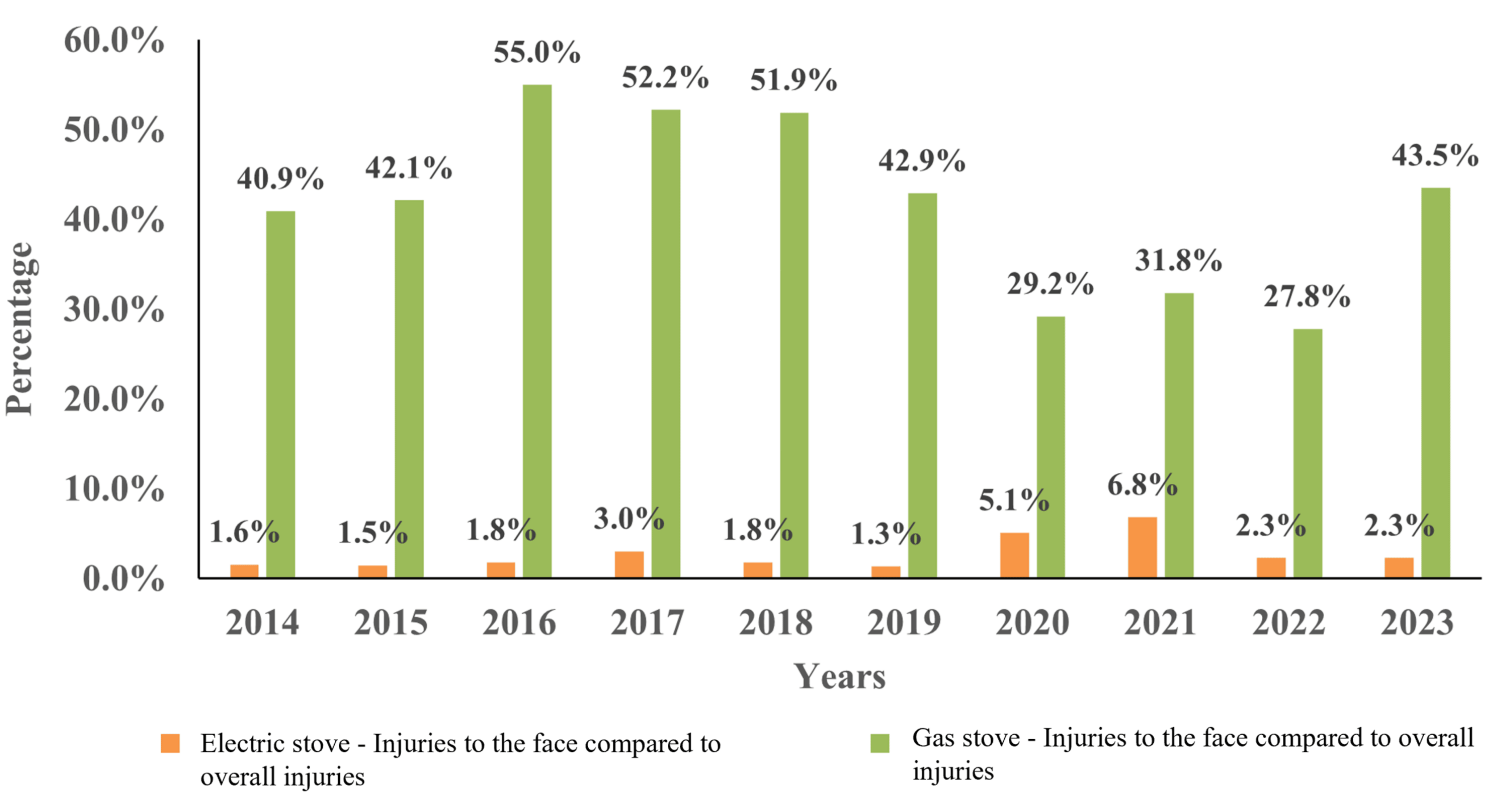
The percentage of electric and gas stove-related burn injuries to the face compared to all types of injuries (e.g., face, hand, lower arm, foot, neck, finger, upper trunk, lower leg, upper arm, head, and lower trunk). The number of electric stove-related burn injuries to the face per year were: 2014 (n=1), 2015 (n=1), 2016 (n=1), 2017 (n=2), 2018 (n=1), 2019 (n=1), 2020 (n=3), 2021 (n=3), 2022 (n=1), and 2023 (n=1). The number of gas stove-related facial injuries were: 2014 (n=9), 2015 (n=8), 2016 (n=11), 2017 (n=12), 2018 (n=14), 2019 (n=12), 2020 (n=7), 2021 (n=7), 2022 (n=10), and 2023 (n=10). For calculating the percentage, burn injuries to the face were compared to all types of injuries (ex: face, hand, lower arm, foot, neck, finger, upper trunk, lower leg, upper arm, head, and lower trunk). Data was gathered from 2014-2023, which includes all age ranges (newborn - 90 years of age).

The highest occurrence of face-related injuries from gas stoves was between 2016 to 2018, peaking in 2016 with 11 cases (55.0%) (Figure 2).

For electric stoves, the highest incidence of face-related injuries occurred in 2020 with three cases (5.1%) and in 2021 with three cases (6.8%), while numbers remained relatively stable in other years. Additionally, there was a notable decrease in face-related injuries from gas stoves between 2016 and 2022, dropping from 11 out of 20 cases (55.0%) to 10 out of 36 cases (27.8%), representing a 27.2% reduction. Overall, face-related injuries were more common with gas stoves compared to electric stoves when all types of stove-related body injuries were considered.

Both figures demonstrated that the newborn to five years age group is the most commonly injured one, regardless of the type of stove involved in the incident. Additionally, injury data related to electric stoves are highly skewed towards the newborn to five years range, with more reported cases than gas stove-related injuries. On average, there were 60 incidents per year involving the electric stove compared to 20 cases with the gas stove. Moreover, gas stove-related burn injuries to the face were significantly higher compared to all types of injuries (e.g., face, hand, lower arm, foot, neck, finger, upper trunk, lower leg, upper arm, head, and lower trunk).

Table 1 presents the frequency of burn injuries categorized by age group and stove type.

**Table 1:** Burn injuries, stratified by age groups and type of stove involved

Age range	Electric stove	Gas stove	Total
0-5	289	26	315
6-11	38	9	47
11-15	15	7	22
16-20	18	9	27
21-25	22	12	34
26-30	25	21	46
31-35	27	16	43
36-40	16	17	33
41-45	21	14	35
46-50	27	8	35
51-55	25	16	41
56-60	17	16	33
61-65	17	15	32
66-70	6	13	19
71-75	5	7	12
76-80	4	4	8
81-85	2	6	8
86-90	2	5	7
Total	576	221	797

A chi-square test of independence revealed a statistically significant association between age group and stove type, χ² (df=17) =139.02, p<0.001, indicating that the distribution of burns varied significantly across these variables. Notably, among children aged 0-5, burns from electric stoves (n=289) were 11 times more frequent than those from gas stoves. Post hoc analyses using standardized residuals indicated that the extreme differences in burn frequency by stove type within the 0-5 age group substantially influenced the Chi-square statistic and the overall statistical significance.

**Figure 3:**
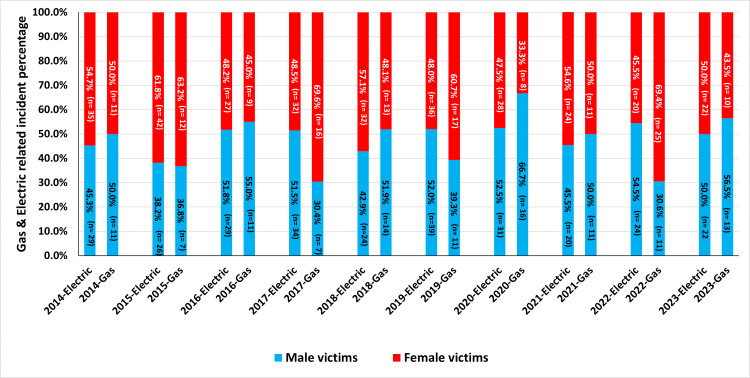
The percentage of burn injuries related to electric and gas stove, as reported in the NEISS database for male and female victims between 2014 and 2023 Data represents annual percentages of burn cases by gender, based on the number of reported incidences across all body locations.

The annual incidence of burn-related injuries from gas and electric stoves with respect to gender from 2014 to 2023, as reported by NEISS, is illustrated in Figure 3.

Among male victims, the highest peak of burn-related injuries from gas stoves occurred in 2020, with 16 cases (66.7%), followed by peaks in 2016 with 11 cases (55.0%) and 2023 with 13 cases (56.5%). Conversely, the lowest incidence of burn-related injuries among male victims from gas stoves was recorded in 2017 with seven cases (30.4%) and in 2022 with 11 cases (30.6%). The highest peak for burn-related injuries from electric stoves among male victims was in 2022, with 24 cases (54.5%). Overall, the trend for burn-related injuries between male and female victims remained relatively stable from 2014 to 2023 (Figure 3).

A chi-square test of independence found substantial deviations between observed and expected burn frequencies by body part and stove type χ² (14, N=612) =265.58, p<0.001 (Table 2).

**Table 2:** Burn frequencies by body part and stove type

Body Part		Electric stove	Gas stove	Total
Face	Count	13	54	67
	Standardized residuals	-12.946	12.946	
Finger	Count	100	7	107
	Standardized residuals	3.921	-3.921	
Foot	Count	14	0	14
	Standardized residuals	1.918	-1.918	
Hand	Count	294	23	317
	Standardized residuals	8.377	-8.377	
Head	Count	0	9	9
	Standardized residuals	-5.966	5.966	
Lower arm	Count	49	19	68
	Standardized residuals	-1.631	1.631	
Lower leg	Count	2	2	4
	Standardized residuals	-1.472	1.472	
Lower trunk	Count	5	2	7
	Standardized residuals	-0.538	0.538	
Mouth	Count	0	1	1
	Standardized residuals	-1.975	1.975	
Neck	Count	0	1	1
	Standardized residuals	-1.975	1.975	
Shoulder	Count	1	1	2
	Standardized residuals	-1.039	1.039	
Upper arm	Count	1	5	6
	Standardized residuals	-3.841	3.841	
Upper leg	Count	1	1	2
	Standardized residuals	-1.039	1.039	
Upper trunk	Count	4	0	4
	Standardized residuals	1.017	-1.017	
Wrist	Count	3	0	3
	Standardized residuals	0.88	-0.88	
Total	Count	487	125	612

A total of 185 stove-related burn cases with unspecified affected body regions were excluded from the statistical analysis. Although electric stoves accounted for the majority of the burn injuries, burns to the face are caused by gas stoves at higher rates than expected by chance. Descriptive statistics indicate that 43% of all gas burns were to the face (i.e., 54 out of 125). Facial burns from electric stoves were rare (i.e., only 15 out of 487 or 3.01%). The standardized residual of +12.95 indicated that this finding was not random. We found much fewer burns to the face from electric stoves than expected and more burns to the face from gas stoves than expected.

## Discussion

As the body’s largest organ and its first line of defense, the skin plays a crucial role in protecting us. Safeguarding this vital barrier is paramount, particularly from everyday risks like kitchen burns. The review of the NEISS database has identified children under the age of five as the most affected group for gas stove-related injuries, with 26 cases (11.7%) out of a total of 221 gas stove-related burns recorded over the nine years covered in this review. With respect to electric stoves, injuries were also most common among the same age group (five years old and below), with 289 cases (50.2%) out of the total 576 electric stove-related burns. In the NEISS database, gas stoves accounted for 221 (27.7%) of the total stove-related burns reported, while electric stoves were responsible for 576 (72.2%) of burns across all age groups. According to the United States Energy Information Administration (EIA), 38.0% of households have a gas stove, while 68.0% of households have an electric stove [9]. When comparing the prevalence of total burns caused by gas stoves and electric stoves, there is a correlation between the type of stove and the households using them: gas stove-related burn injuries represented 221 cases (27.7%) among 38% of households using gas stoves, while electric stove-related burn injuries accounted for 576 cases (72.2%) among 68% of households using electric stoves [9].

Although there is no significant difference in the prevalence of gas versus electric stove burns relative to the proportion of gas and electric stoves found in US households, our results show a notable increase in facial burns associated with gas stoves. Over the nine years covered in the review, 92 of the 221 gas stove burns (41.7%) involved the face. Conversely, only 16 of the 576 electric stove burns (2.7%) were facial burns. The increase in facial burns from gas stoves can be attributed to the ignition of the open flame, causing long-distance flash burns [10,11]. Flash burns occur when there is an excess amount of natural gas in the air, leading to rapid combustion that results in burns on the upper extremity, torso, and face [12]. On the other hand, the data illustrated that electric stoves typically produce burns through direct contact, primarily affecting the hands and fingers.

When comparing the treatment protocols for burns caused by gas versus electric stoves, it is crucial to recognize that both types of burns are classified as thermal burns. Thermal burns occur due to external heat sources that raise the skin's temperature, resulting in tissue cell death [13]. The Mayo Clinic advises seeking medical attention for burns that blister, cover a large area, or affect critical regions such as the face. The treatment of burns is further determined by their severity: first-degree burns affect only the outer layer of the skin, second-degree burns extend to the underlying layers and often cause blistering, and third-degree burns penetrate all skin layers, potentially affecting underlying tissues [13]. Specific treatments for thermal burns include cooling the burn under running water, applying antibiotic ointments, covering the burn with a sterile gauze bandage, and taking pain relievers. Severe burns may require professional medical interventions such as intravenous fluids, antibiotics, and sometimes surgery [14]. Consequently, there is no significant difference in the treatment protocols for burns caused by gas versus electric stoves, underscoring the importance of prompt and appropriate treatment for all thermal burn injuries to prevent complications and promote healing.

Most stove-related burns are considered minor and are typically managed at home or treated as outpatient cases. Basic first aid measures, such as cooling the burn under running water, applying antibiotic ointments, and using over-the-counter pain relievers, are usually sufficient for these injuries. However, patients are transferred and treated at specialized burn centers based on key factors, including the extent of body surface area burned, depth of the burn, age, and other significant medical history. This triage system ensures that those with more severe or complicated burns receive the necessary specialized care while allowing minor burns to be effectively managed outside of a hospital setting [15].

Age significantly influences the likelihood of seeking medical care for burn injuries, with parents showing a markedly higher tendency to bring young children to the emergency department (ED) compared to older children or adults who may delay or avoid seeking care for themselves. This pattern likely reflects the unique physiological vulnerabilities of young children and the heightened parental concern for their well-being. Clinically, a burn involving just 10% of a child's total body surface area (TBSA) is classified as a major injury, whereas adults typically require burns covering 15-20% TBSA to fall under the same classification [16]. Such differences underscore the disproportionate impact burns can have on children, leading to more severe outcomes and more frequent ED visits among those aged newborn to five years, the most commonly affected age group in burn injury cases. To mitigate these risks, kitchen appliance manufacturers could integrate advanced sensor-based safety mechanisms. For example, gas stoves could be equipped with proximity sensors capable of detecting a large flesh-like surface, such as the face, within a hazardous distance, triggering an automatic shut-off to prevent ignition-related burns. Likewise, electric stoves could benefit from tactile sensors calibrated to recognize small, keratin-like contact points (e.g., fingernails or fingertips), enabling a rapid power shutdown in response to unintended touch. Such innovations could significantly reduce the frequency and severity of stove-related injuries, particularly among vulnerable populations such as young children and older adults.

Healthcare providers play a vital role in burn injury prevention by educating patients about common causes, such as contact with hot surfaces and the risks of leaving children unsupervised in the kitchen. They can offer guidance on using protective gear when operating gas or electric stoves and provide essential first aid training to ensure immediate and effective care when burns occur. Through proactive safety education and collaborative care, individuals can take meaningful steps to protect their skin and preserve its critical role as a barrier against injury and infection.

Notably, the NEISS review demonstrates a post-COVID-19 decline in gas-stove-related burns, juxtaposed with a modest rise in electrical burns. This pattern reflects evolving household behaviors and external socio-economic pressures during the pandemic. Although direct surveillance of gas-stove injuries post-pandemic remains scant, attention to the indoor pollution risks of gas appliances, heightened during the COVID-19 pandemic, may have prompted behavioral modifications [17]. Indoor air quality concerns, notably about pollutants like NO₂, have increased scrutiny of gas cooking devices, encouraging households to improve ventilation or transition to electric or induction stoves, potentially contributing to fewer gas-related burn injuries [17].

Thermal burns, which constitute approximately 86% of admissions to specialized burn centers, disproportionately affect young children due to their thinner skin, limited motor coordination, and increased exposure to domestic hazards such as hot liquids, heated surfaces, and open flames [14]. This heightened susceptibility, coupled with the potential for life-altering complications, often compels caregivers to seek immediate and specialized medical attention. As a result, there is a critical demand for pediatric-focused burn care services, including targeted prevention strategies and tailored rehabilitation protocols [18,19].

In order to address our study’s limitations, future research should seek increased sample size, ensuring balanced representation across age groups and genders, and integrate socioeconomic variables that may influence burn incidence and access to care. It is also important to analyze data from countries with a comparable standard of living, as differences in infrastructure, healthcare access, and household environments may significantly influence both the epidemiology of burns and the effectiveness of prevention strategies. There is a possibility that the number of burns in older age groups is underreported due to lower number of victims seeking medical care. For instance, studies show that high-income countries have lower mortality despite higher rates of burn injury; the study highlights how low- to middle-income countries could be experiencing increased mortality, despite decreased rates of burn injury due to lack of resources [20]. Evaluating data from economically and structurally similar settings will ensure more valid comparisons and guide interventions that are both evidence-based and contextually appropriate.

Additionally, advancements in household safety, such as the incorporation of stove knob covers, automatic flame-failure shutoff devices, induction cooktops that remain cool to touch, and heat-resistant barrier guards, should be evaluated for their role in reducing pediatric burns. Public health initiatives could integrate parent-focused safety campaigns that emphasize active supervision, establishing a “no-go” safety zone around cooking areas, and routine testing of smoke and carbon monoxide alarms. Community-level interventions might include subsidized distribution of child-safety equipment to low-income households and collaboration with appliance manufacturers to incorporate injury-prevention features into standard stove designs. Access to comprehensive medical records and the development of standardized, evidence-based treatment guidelines will further support optimized care for pediatric burn patients and enable more effective interventions at both clinical and community levels.

## Conclusions

According to NEISS data, children aged zero to five years are at the highest risk of both gas and electric stove-related injuries compared to other age groups. The overall trend for burn-related incidences between male and female victims remained relatively stable from 2014 to 2023. There is a clear distinction in the primary regions of the body affected by different stove types: gas stoves are more frequently associated with facial burns, likely due to sudden flare-ups or direct exposure to open flames, while electric stoves tend to cause injuries to the hands, often from contact with hot surfaces or cookware. These patterns suggest the need for tailored safety interventions based on stove type.
